# Skeletonized Handmade Valved Conduit: A Novel Approach for Transannular Repair in Absent Pulmonary Valve Syndrome

**DOI:** 10.1016/j.atssr.2025.01.023

**Published:** 2025-02-26

**Authors:** Hiroki Ito, Keiichi Hirose, Kisaburo Sakamoto, Akio Ikai

**Affiliations:** 1 Department of Cardiovascular Surgery, Mt. Fuji Shizuoka Children’s Hospital, Shizuoka, Japan; 2 Pulmonary Hemodynamics Research Division, Department of Clinical Research, Research Support Center, Shizuoka General Hospital, Shizuoka, Japan

## Abstract

Absent pulmonary valve syndrome in neonates and infants presents challenges due to airway compression from dilated pulmonary arteries and limited anatomical space for right ventricular outflow tract (RVOT) reconstruction. This study introduces a half-skeletonized handmade valved conduit for transannular repair in a 6-month-old boy with absent pulmonary valve syndrome, trisomy 21, and laryngomalacia. The conduit, made from expanded polytetrafluoroethylene, provided effective RVOT reconstruction while minimizing airway obstruction. Postoperative imaging confirmed bronchial decompression, and echocardiography showed sustained valve function at 1 year. This technique addresses homograft limitations and may have broader applicability in congenital heart defects requiring RVOT reconstruction.

The surgical management of absent pulmonary valve syndrome (APVS) in neonates and infants is challenging due to the interplay of hemodynamic instability and airway compression caused by dilated pulmonary arteries. Traditional right ventricular outflow tract (RVOT) reconstruction techniques often rely on homografts, which are frequently unavailable or not appropriately sized for this population.^1^^,^^2^ Furthermore, these methods may exacerbate preexisting bronchial compression, posing risks to respiratory function.

This study introduces a novel solution: a half-skeletonized handmade tricuspid valved conduit, designed to address the dual challenges of space limitation and bronchial compression.^3^^,^^4^ By providing a customizable approach, this technique ensures effective RVOT reconstruction and minimizes postoperative complications.

## Technique

**Figure 1:**
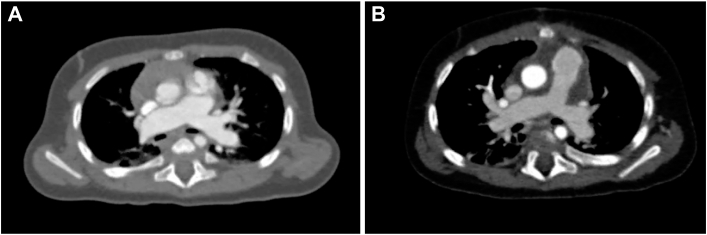
(A) Preoperative computed tomographic scan shows severe bronchial compression due to dilated central pulmonary arteries. (B) Postoperative computed tomographic scan demonstrates significant resolution of bronchial compression after transannular repair with a half-skeletonized handmade valved conduit.

A 6-month-old boy weighing 7.5 kg was diagnosed in utero with double-outlet right ventricle (DORV) and absent pulmonary valve syndrome (APVS) and was monitored after birth. The patient also had trisomy 21 and laryngomalacia and experienced gradual and progressive respiratory distress. Computed tomography revealed a dilated central pulmonary artery (PA) compressing the main bronchi on both sides (Figure 1A).^2^

**Figure 2:**
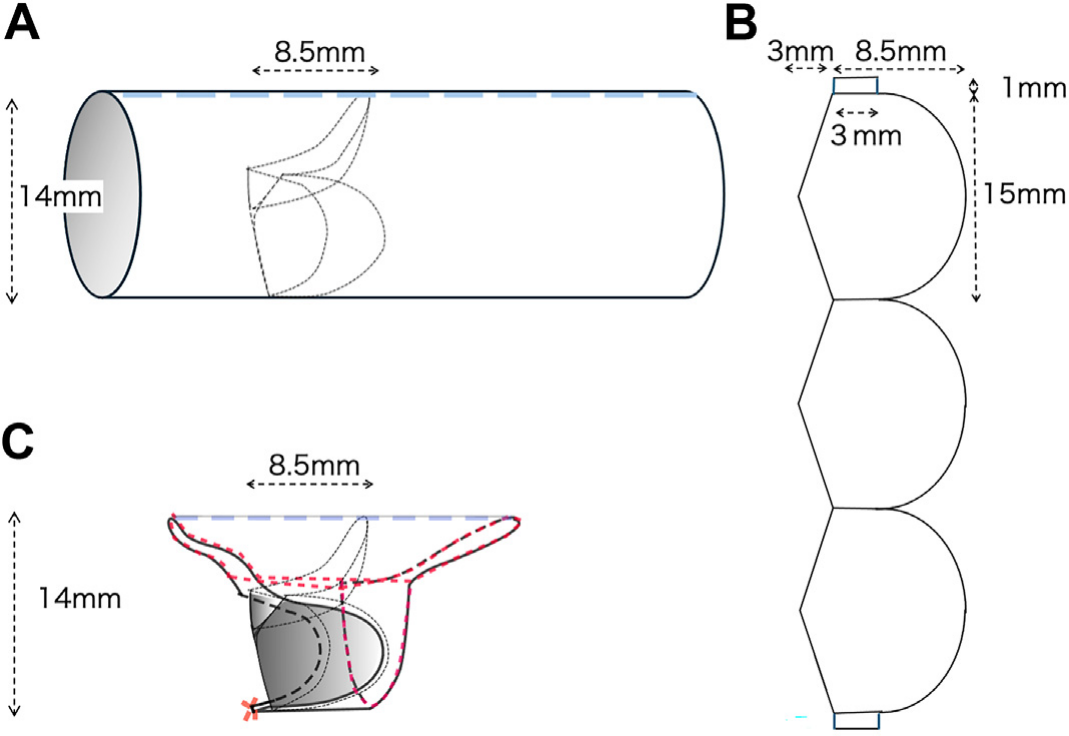
Schematic of conduit design and modification. (A) Basic design of the handmade tricuspid valved conduit with a cusp height set at two-thirds of the graft diameter (14 mm). (B) Detailed cusp structure and dimensions used in the handmade design. (C) Half-skeletonized conduit design illustrates the resection of posterior wall segments to reduce bronchial compression while preserving functional valve geometry. The red dashed lines indicate the suture lines used for securing the conduit.

In the operating room, a handmade tricuspid valved conduit with a diameter of 14 mm was fabricated using expanded polytetrafluoroethylene (ePTFE). Our valve design featured a cusp height of 8.5 mm, which is two-thirds of the graft diameter (Figures 2A, 2B). Cardiopulmonary bypass was initiated, the ascending aorta was cross-clamped, and the heart was arrested with crystalloid cardioplegic solution. A transannular incision was then made. A doubly committed ventricular septal defect (VSD) was observed with no space between the pulmonary annulus and the superior edge of the VSD. A pulmonary valve remnant was identified.

The VSD was closed with an ePTFE patch using a 6-0 polypropylene running suture. The dilated bilateral PAs were plicated up to the pulmonary hilum to a diameter of ∼8 mm, and the posterocaudal PA wall was resected. To avoid further bronchial compression and stenosis of the PA bifurcation, the idea of transecting the main PA and inserting a valved conduit between the dilated right ventricle and PA was abandoned.

### Distal Anastomosis

To maintain valve geometry and prevent distortion, the posterocaudal margin of the conduit is secured with precise continuous sutures, ensuring proper leaflet coaptation and function. At the distal anastomosis, the strut between the 2 posterior cusps is secured to the central PA wall. Owing to the extremely short main PA and the dilated left and right PAs in APVS, the strut is attached beyond the main PA component at the bifurcation of the left and right PAs. The struts on both sides of the anterior valve are carefully aligned, and the graft is trimmed into a half-dome shape to provide sufficient margin for suturing. The stiffness of the ePTFE graft complements the elasticity of the native PA, ensuring a secure fit that maintains stability at the anastomosis and preserves valve function (Figure 2C).

### Postoperative Outcome

The patient was weaned from cardiopulmonary bypass with normal inotropic support. The bypass time was 257 minutes, and the aortic cross-clamp time was 173 minutes (Video 1). Postoperatively, the patient was extubated on day 1 and discharged on day 10. Echocardiography revealed mild regurgitation with a peak velocity of 1.9 m/s in the pulmonary valve at discharge. Bronchial decompression was confirmed by postoperative computed tomography (Figure 1B). At 1 year, echocardiography showed mild regurgitation with a peak velocity of 2.6 m/s, and catheterization revealed a systolic right ventricular pressure of 42 mm Hg and a mean PA pressure of 19 mm Hg (Video 2).

## Comment

The half-skeletonized handmade valved conduit offers an effective alternative for managing APVS, particularly in cases of severe airway compression or limited operative space. Although standard nonskeletonized ePTFE conduits are sufficient in many cases, markedly dilated PAs encroaching on the bronchi may require a more tailored approach. Resecting the posterior wall segments reduces the bulk of the conduit, allowing for direct anastomosis to the pulmonary annulus. This technique ensures a snug fit, maintains valve function, and provides a practical solution for anatomically challenging scenarios. In the present case, postoperative imaging confirmed effective bronchial decompression, and echocardiography at 1 year demonstrated excellent valve performance with only mild regurgitation, which was deemed clinically acceptable.^2^^,^^3^

Conventional RVOT reconstruction techniques often use homografts, which are challenging to obtain in appropriately sized options for neonates and infants. Additionally, bovine jugular vein grafts, although available, are associated with a higher profile, which can be less suitable in certain cases. In contrast, the described technique uses readily available ePTFE materials, offering a customizable and practical solution that addresses these limitations.^5^^,^^6^ Furthermore, the design of the conduit, with a cusp height proportional to the graft diameter, mimics natural valve dynamics, ensuring effective hemodynamic function in a confined space.^4^

Although this approach was developed specifically for APVS, its customizable design suggests potential applicability to other congenital heart defects requiring pulmonary valve replacement or RVOT reconstruction. Conditions involving RVOT obstruction or valve dysfunction could benefit from this technique’s ability to accommodate unique anatomical challenges.^5^

This method addresses critical limitations of existing techniques, offering a reliable and customizable solution for APVS. Additionally, its flexibility positions it as a promising strategy for pulmonary valve interventions in a broader range of congenital heart conditions. Future studies are warranted to evaluate long-term efficacy and broader clinical applications.

## Declaration of Generative AI and AI-Assisted Technologies in The Writing Process

During the preparation of this work, the authors used ChatGPT (OpenAI) in order to enhance language and readability. After using this tool, the authors reviewed and edited the content as needed and take full responsibility for the content of the publication.
